# The human coronary vasodilatory response to acute mental stress is mediated by neuronal nitric oxide synthase

**DOI:** 10.1152/ajpheart.00745.2016

**Published:** 2017-06-23

**Authors:** Sitara G. Khan, Narbeh Melikian, Husain Shabeeh, Ana R. Cabaco, Katherine Martin, Faisal Khan, Kevin O’Gallagher, Philip J. Chowienczyk, Ajay M. Shah

**Affiliations:** ^1^Department of Cardiology, Faculty of Life Sciences & Medicine, British Heart Foundation Centre, King’s College London, London, United Kingdom; and; ^2^Department of Clinical Pharmacology, Faculty of Life Sciences & Medicine, British Heart Foundation Centre, King’s College London, London, United Kingdom

**Keywords:** mental stress, coronary, endothelial function, nitric oxide, human

## Abstract

Mental stress-induced ischemia approximately doubles the risk of cardiac events in patients with coronary artery disease, yet the mechanisms underlying changes in coronary blood flow in response to mental stress are poorly characterized. Neuronal nitric oxide synthase (nNOS) regulates basal coronary blood flow in healthy humans and mediates mental stress-induced vasodilation in the forearm. However, its possible role in mental stress-induced increases in coronary blood flow is unknown. We studied 11 patients (6 men and 5 women, mean age: 58 ± 14 yr) undergoing elective diagnostic cardiac catheterization and assessed the vasodilator response to mental stress elicited by the Stroop color-word test. Intracoronary substance P (20 pmol/min) and isosorbide dinitrate (1 mg) were used to assess endothelium-dependent and -independent vasodilation, respectively. Coronary blood flow was estimated using intracoronary Doppler recordings and quantitative coronary angiography to measure coronary artery diameter. Mental stress increased coronary flow by 34 ± 7.0% over the preceding baseline during saline infusion (*P* < 0.01), and this was reduced to 26 ± 7.0% in the presence of the selective nNOS inhibitor *S*-methyl-l-thiocitrulline (0.625 µmol/min, *P* < 0.001). Mental stress increased coronary artery diameter by 6.9 ± 3.7% (*P* = 0.02) and 0.5 ± 2.8% (*P* = 0.51) in the presence of *S*-methyl-l-thiocitrulline. The response to substance P did not predict the response to mental stress (*r*^2^ = −0.22, *P* = 0.83). nNOS mediates the human coronary vasodilator response to mental stress, predominantly through actions at the level of coronary resistance vessels.

**NEW & NOTEWORTHY** Acute mental stress induces vasodilation of the coronary microvasculature. Here, we show that this response involves neuronal nitric oxide synthase in the human coronary circulation.

Listen to this article’s corresponding podcast at http://ajpheart.podbean.com/e/nnos-and-coronary-flow-during-mental-stress/.

## Abstract (precis)

Acute mental stress induces vasodilation of the coronary microvasculature. Here, we show that this response involves neuronal nitric oxide synthase in the human coronary circulation.

acute mental stress can impact on cardiovascular morbidity and mortality, triggering myocardial infarction, ventricular arrhythmia, and left ventricular failure (11). Analogous to exercise-induced stress, mental stress can exert detrimental effects due to suboptimal coupling between myocardial oxygen demand and blood flow (31). The physiological cardiovascular response to mental stress mirrors that to other sympathetic stimuli and includes a catecholamine-driven increase in heart rate, blood pressure, and cardiac contractility (17). Coronary blood flow increases in parallel to the higher myocardial O_2_ demand, mediated mainly by reduction in coronary vascular resistance. Factors identified to mediate coronary vasodilator reserve include Ca^2+^-activated K^+^ channels (27), adenosine, and nitric oxide (NO) (45). The vasomotor response to mental stress can be attenuated or even reversed in the presence of coronary artery disease, a condition that features endothelial dysfunction and reduced NO availability (9, 41, 43).

Until relatively recently, it was generally assumed that the NO responsible for mediating local increases in blood flow was generated by endothelial NO synthase (eNOS) expressed in endothelial cells (10, 16). However, studies using intra-arterial infusion of a selective neuronal NO synthase (nNOS) inhibitor show that local nNOS-derived NO is a major contributor to the basal regulation of microvascular tone and blood flow in the human forearm and coronary circulations (32, 33). It was also found that local nNOS is involved in mental stress-induced forearm vasodilatation in healthy humans. In the present study, we investigated the role of nNOS in the changes in coronary blood flow during acute mental stress in humans.

## METHODS

The study was conducted in accordance with the Declaration of Helsinki and received ethical approval from the local Research and Ethics Committee. All participants provided written informed consent. Eleven patients (6 men and 5 women, mean age: 58 ± 14 yr) undergoing elective diagnostic coronary angiography at King’s College Hospital (London, UK) were included in the study. Patients had to have at least one angiographically unobstructed coronary artery as the study vessel. Those with a history of coronary disease, myocardial infarction, left ventricular impairment, dysrhythmia, or renal impairment were excluded. Participants abstained from food for at least 6 h before cardiac catheterization and from all medication (except aspirin) on the day of the procedure.

Standard diagnostic coronary angiography was performed in a quiet, temperature-controlled cardiac catheterization laboratory with digital cineangiography. After completion of the diagnostic procedure, a 6-F guide catheter was positioned in the study artery, and a 0.014-in. intracoronary Doppler wire (FloWire, Volcano Therapeutics, Rancho Cordova, CA) was advanced into a proximal segment that was free from side branches. The Doppler wire was interfaced with a real-time spectral analysis system (ComboMap Pressure and Flow system, Volcano Therapeutics) to record the average peak velocity (APV) of blood flow. APV recordings were taken before coronary angiography at each stage of the protocol, which was performed using non-ionic contrast medium (Omnipaque, GE Healthcare) and without altering the angle of projection during the study. Offline analysis was performed using an automated quantitative coronary edge detection system (Philips) to measure changes in epicardial coronary artery diameter in a 2.5- to 5-mm length segment of vessel, ~2.5 mm distal to the tip of the Doppler wire. Coronary flow was derived from the APV and arterial diameter (12).

All drugs were infused via the guide catheter into the study artery at a rate of 2 ml/min. The selective nNOS inhibitor *S*-methyl-l-thiocitrulline (SMTC) was obtained from Merck Millipore and was prepared to good medical practice standards for human use in a nationally accredited pharmaceutical manufacturing facility. Substance P was obtained from Bachem (Bubendorf, Switzerland), and isosorbide dinitrate (ISDN) was obtained from Schwarz Pharma (Watford, UK).

**Fig. 1.**
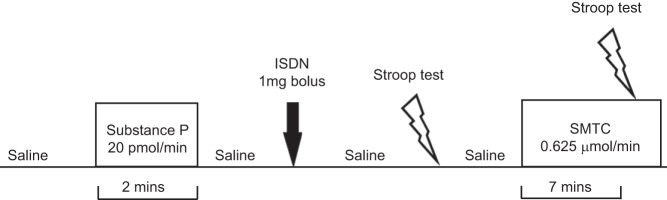
Schematic representation of the infusion protocol. Average peak velocity, coronary angiography, blood pressure, heart rate, and ECG were recorded at each stage. ISDN, isosorbide dinitrate; SMTC, *S*-methyl-l-thiocitrulline.

A schematic representation of the protocol is shown in Fig. 1. Measurements began once a steady baseline was achieved during intracoronary infusion of 0.9% saline. Substance P was then infused for 2 min at 20 pmol/min, a dose that elicits eNOS-mediated, endothelium-dependent vasodilation without inducing systemic effects (24). After a washout period with normal saline, a 1-mg ISDN bolus was administered to assess endothelium-independent vasodilatation (32). After a further saline washout, the Stroop color-word test was performed to elicit mental stress (15). This was followed by a recovery period after which SMTC (0.625 µmol/min) was infused for 7 min. This dose of SMTC has previously been shown to provide selective inhibition of nNOS in the coronary circulation in a similar patient population (32). SMTC infusion was continued while the Stroop test was repeated. In two patients, a second Stroop test was performed during saline instead of SMTC infusion to confirm that it evoked a reproducible increase in blood flow. Aortic pressure, heart rate, and APV were recorded at baseline and after each phase of the protocol, and coronary angiography was performed to quantify coronary diameter. The ECG was continuously monitored.

### 

#### Statistical analyses.

Subject characteristics are summarized as means ± SD; other results are presented as means ± SE. Vasodilator/constrictor responses were analyzed both as absolute change in coronary flow and as the percent change from the immediately preceding baseline. Responses were compared by ANOVA for repeated measures with adjustment for baseline values of coronary blood flow where appropriate [ANOVA/analysis of covariance (ANCOVA)]. Bonferroni correction for multiple comparisons was applied. All tests were two-tailed, and differences were considered significant at *P* < 0.05.

## RESULTS

Baseline subject characteristics are shown in Table 1. The indications for coronary angiography included chest pain (7 subjects), dyspnea (2 subjects), nonsustained ventricular tachycardia on Holter monitoring (1 subject), and preoperative assessment in valvular disease (1 subject). Four subjects underwent functional testing for ischemia (either stress echocardiography, myocardial perfusion scintigraphy, or exercise treadmill testing), which was positive in three cases. Ten subjects had all three coronary arteries smooth and unobstructed; the 11th subject had minor irregularities (<10% stenosis) in all arteries. None of the subjects developed adverse reactions, symptoms, or ECG changes of ischemia during the infusions or mental stress.

**Table 1.** Baseline characteristics of patients

Characteristic	Value
*N*	11
Age, yr	58 ± 14
Sex, men/women	6/5
Hypertension, *n*	7
Diabetes mellitus, *n*	2
Hypercholesterolemia, *n*	8
Smoker, *n*	0
Body mass index, kg/m^2^	28.2 ± 3.2
Medication, *n* (%)	
Angiotensin-converting enzyme inhibitor/angiotensin receptor blocker	4 (36)
β-Blockers or Ca^2+^ channel blocker	5 (45)
Statins	2 (18)
Study artery, *n*	
Left anterior descending coronary arter	7
Circumflex artery	3
Right coronary artery	1

Values are means ± SD; *n*, no. of subjects.

### 

#### Effect of mental stress and SMTC on coronary flow.

The hemodynamic responses and changes in coronary blood flow and coronary conductance after mental stress are shown in Table 2. The changes in heart rate and blood pressure during mental stress were minimal and similar with or without SMTC.

**Table 2.** Hemodynamic responses during mental stress with or without SMTC

	Saline		SMTC	
	Baseline	Stress	Baseline	Stress
Heart rate, beats/min	59 ± 3.7	60 ± 3.9	55 ± 3.0	62 ± 4.9
Systolic BP, mmHg	117 ± 3.7	120 ± 6.0	114 ± 4.0	119 ± 5.4
Mean BP, mmHg	84 ± 1.4	83 ± 2.7	81 ± 3.0	84 ± 3.5
Diastolic BP, mmHg	68 ± 2.3	64 ± 2.5	65 ± 3.9	67 ± 4.3
Coronary blood flow, ml/min	38 ± 3.9	52 ± 5.4†	31 ± 4.0	40 ± 4.5†‡
Coronary conductance, units	0.49 ± 0.04	0.61 ± 0.08*	0.42 ± 0.06	0.53 ± 0.05*‡

Values are means ± SE. SMTC, *S*-methyl-l-thiocitrulline; BP, blood pressure.

* *P* < 0.05 and

† *P* < 0.01 vs. the preceding baseline.

‡ Significant interaction between groups.

**Fig. 2.**
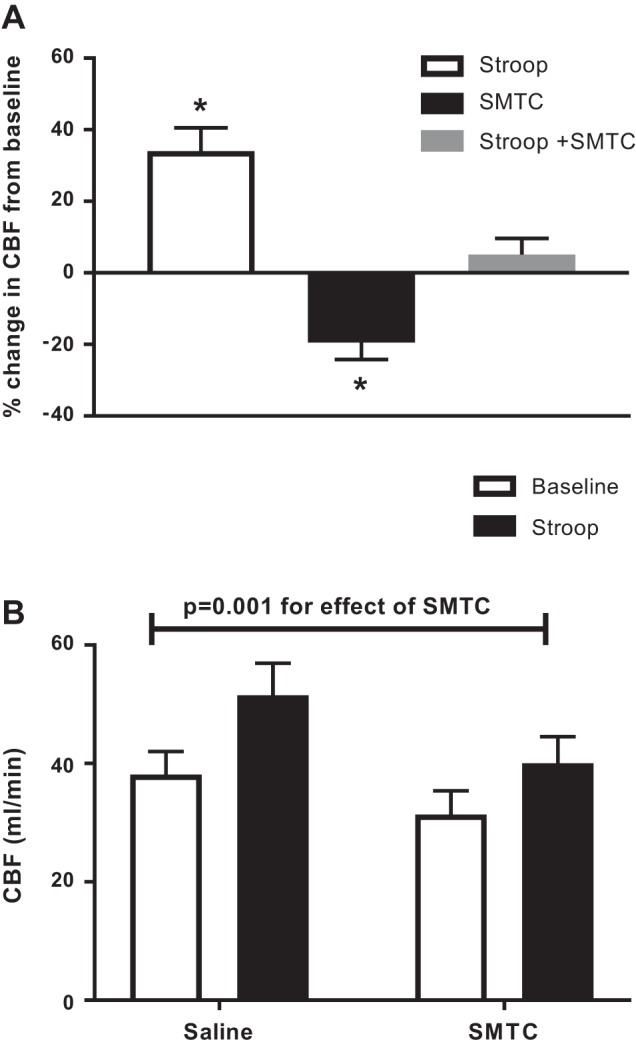
Effect of SMTC on the mental stress-induced increase in coronary blood flow (CBF). *A*: percent change in CBF with the Stroop test during saline infusion (Stroop), SMTC, and the Stroop test during SMTC infusion. Paired *t*-test was between CBF during the preceding baseline and intervention. *P < 0.01 compared with the preceding baseline. *B*: absolute values for CBF under the different study conditions. *Significant interaction between groups by repeated-measures ANOVA. Values are means ± SE; *n* = 11.

Mental stress increased coronary flow by 34 ± 7.0% (*P* < 0.01; Fig. 2*A*). SMTC reduced basal coronary flow by 20 ± 4.7% (*P* = 0.01), consistent with previous work (32, 33). It also significantly attenuated the vasodilator effect of mental stress, reducing the coronary flow response to a 26 ± 7.0% increase compared with the preceding baseline (Fig. 2*B*).

Substance P increased basal coronary flow by 24 ± 8.0% (*P* = 0.01). There was no significant correlation between the response to substance P and that to mental stress (*r*^2^ = −0.22, *P* = 0.83). ISDN increased coronary flow in all subjects (mean increase of 119 ± 40%), indicating an intact smooth muscle response to NO.

#### Effect of mental stress and SMTC on epicardial coronary diameter.

**Fig. 3.**
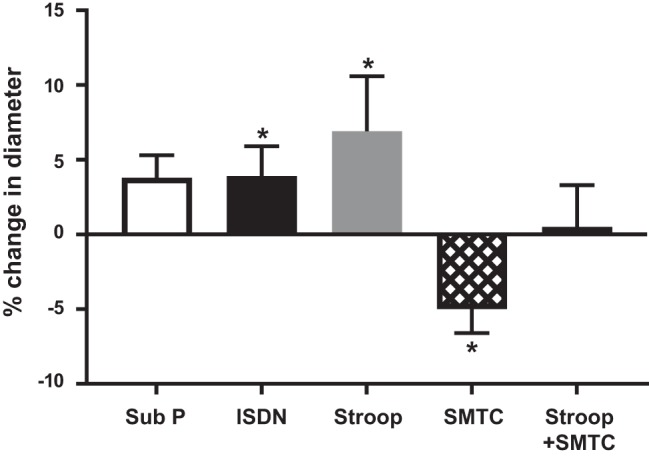
Changes in epicardial coronary artery diameter in response to interventions. Percent changes from the preceding baseline are shown. Sub P, substance P. Values are means ± SE; *n* = 11. Paired *t*-test was between diameter during the preceding baseline and intervention. *P < 0.05 vs. the preceding baseline.

Mental stress increased coronary artery diameter by 6.9 ± 3.7% (*P* = 0.02; Fig. 3). SMTC reduced coronary artery diameter by 5.1 ± 1.6% (*P* < 0.01) and abolished the mental stress-induced increase to 0.5 ± 2.8% (*P* = 0.98 compared with the preceding baseline). ISDN increased diameter by 3.9 ± 2.0% (*P* = 0.01), and substance P increased it by 3.1 ± 1.6% (*P* = 0.07). There was no significant correlation between the percent change in coronary diameter and percent change in coronary blood flow in response to acute mental stress (*r*^2^ = 0.19, *P* = 0.15).

## DISCUSSION

NO plays an important role in the regulation of vasomotor tone in the human coronary circulation, both during resting conditions and under situations of increased metabolic demand. At rest, it maintains a state of tonic vasodilation in resistance vessels (21) and conduit epicardial arteries (22). After stimuli such as mental stress and cold pressors (3, 9, 13, 26), NO mediates dynamic changes in vascular tone that antagonize catecholamine-mediated vasoconstriction.

Until relatively recently, investigations of the role of NO in regulating coronary vascular tone in humans relied mainly on the use of the nonselective NOS inhibitor *N*^G^-monomethyl-l-arginine (l-NMMA) or of endothelium-dependent agonists, such as ACh, substance P, and fluid shear stress (14, 21, 36–38). Based on such studies, eNOS expressed in the endothelium was assumed to be the main source of local NO that regulated vascular tone. However, experimental animal studies (25) have indicated that nNOS expressed in perivascular nerves or the vascular smooth muscle might also regulate local vessel tone. In line with this, nNOS is reported to be expressed in human coronary artery smooth muscle cells (18) and coronary perivascular nerves in rats and dogs (34, 44).

In first-in-human studies with the nNOS-selective inhibitor SMTC, our laboratory previously demonstrated that basal blood flow in the human coronary circulation in vivo is under tonic regulation by local nNOS-derived NO (32). In these experiments, we found that SMTC had no effect on endothelium-dependent vasodilation induced by substance P, which was, however, inhibited by l-NMMA. Similarly, local SMTC infusion into the forearm circulation of healthy human subjects reduced basal blood flow in an l-arginine-dependent manner but without affecting the vasodilator response to ACh (33). Furthermore, we found that mental stress-induced increases in forearm blood flow in healthy men were significantly blunted by local intra-arterial infusion of SMTC, suggesting that local nNOS was involved in this setting. Previous work in animals has shown that nNOS-dependent effects on vessel tone vary significantly by vascular bed (10, 19, 23), and so it was important to establish whether nNOS is involved in mental stress-induced vasodilatation in the human coronary circulation.

The novel finding of this study is that nNOS is responsible for mediating mental stress-induced vasodilation in the resistance vasculature of the human coronary circulation. The changes in coronary flow and conductance were not related to significant changes in systemic hemodynamics, suggesting that they represented a direct vasodilator action of nNOS-derived NO and not effects secondary to altered myocardial O_2_ demand. This role of nNOS contrasts to pacing-induced increases in coronary blood flow, which we recently reported were mediated by eNOS-derived NO (35). The activation of eNOS during pacing is likely to involve increased endothelial shear stress, whereas nNOS activation during mental stress may involve perivascular nitrergic nerves in the coronary vessel wall and their autonomic activation (2, 5).

The baseline response to mental stress in the present study was variable, ranging from a 1−77% increase in blood flow, similar to our laboratory’s previous observations in the forearm. Previous studies on mental stress-induced vasodilation in humans have reported average increases ranging from a 55% increase in blood flow (9) to a 10% increase in flow (43), which may be related to the differing characteristics of study participants. Specific factors that could have influenced the vascular response to stress in our study include vasoactive medications, body mass index, and diabetes. Local characteristics of the vessel studied could also have influenced the response, although we selected vessels that were angiographically free of stenosis. The use of different techniques to elicit mental stress may also contribute to variation in responses. In the present study, SMTC reduced basal coronary flow, as previously documented (32, 33), and substantially attenuated the mental stress-induced increase in blood flow. The degree of attenuation in stress-induced vasodilation is similar to that previously observed in the forearm (20), although it should be noted that subject characteristics were significantly different between these studies. The present study by necessity was undertaken in patients undergoing diagnostic coronary angiography and, therefore, included older patients with multiple risk factors for coronary disease and on oral medications.

Impaired mental stress-induced increases in flow have been reported in coronary artery disease (9, 31), metabolic syndrome (8), and nonflow-limiting atherosclerosis (43). Interestingly, we found little correlation between the response to substance P and the response to mental stress, suggesting that eNOS-mediated vasodilator function may not be the major determinant of the mental stress response. It is possible, therefore, that the variations in the mental stress response could, in part, be explained by the presence of nNOS dysfunction in these conditions (7, 30, 39). Potential underlying mechanisms include nNOS uncoupling, which has been documented in conditions of oxidative stress, such as atherosclerosis, and increased levels of the endogenous NO synthase inhibitor asymmetric dimethylarginine, which has been observed in conditions such as diabetes and hypertension (29).

Numerous studies have been performed to explore the impact of stress on patients with coronary artery disease, and a recent meta-analysis found that mental stress-induced ischemia is associated with an approximately doubled risk of cardiac events (myocardial infarction, revascularization, and unstable angina) and total mortality (42). The finding of reduced coronary blood flow during mental stress has also been shown to be a predictor of daily ischemia in patients with coronary artery disease, independent of exercise-induced ischemia (4).

Little is known about the long-term outcomes associated with impaired mental stress-induced vasodilation in people who have been diagnosed with “normal coronary arteries.” There are some important considerations to bear in mind. First, it is now established that coronary angiography can underestimate the degree of plaque burden compared with postmortem histology (40). Second, a degree of “visual-functional” mismatch occurs on coronary angiography, which can under- or overestimate a plaque’s functional severity in up to 40% of cases (28). Third, it is increasingly understood that the majority of acute coronary syndromes arise from plaques that are not necessarily flow limiting in size but share vulnerable characteristics that ultimately lead to their rupture (6). Indeed, Yeung et al. (43) found that mental stress triggers vasoconstriction at the site of both obstructive and nonobstructive plaques in epicardial arteries, with an accompanying decrease in myocardial blood flow. Additionally, Arrighi et al. (1) found that mental stress caused increased coronary resistance and impaired myocardial blood flow in regions subtended by vessels without significant stenosis. In the present study, we observed nNOS-dependent changes in epicardial coronary artery diameter, as well as coronary blood flow, in response to acute mental stress. These effects were not correlated with each other, suggesting that they may both reflect direct effects of nNOS, but we cannot exclude the possibility that the changes in epicardial diameter might be secondary to the changes in blood flow. Given the identification of nNOS as a mediator of stress-induced coronary flow in individuals with angiographically normal arteries, it would be of interest to examine its function in patients with established coronary disease.

In conclusion, this study identified nNOS as the major NO synthase isoform responsible for mediating the vasodilator response to mental stress in the human coronary circulation. Due to the complex interplay that exists between mental stress and vasodilator function in the coronary circulation, dysfunctional blood flow responses to mental stress may contribute to the development of stress-induced myocardial ischemia, irrespective of the presence of angiographically significant coronary artery disease.

### 

#### Study limitations.

We estimated coronary blood flow from flow velocity rather than using thermodilution, but flow velocity has been widely used in studies of coronary vascular function. All of the participants in our study either had risk factors for vascular endothelial dysfunction and/or were on vasoactive medications, so that the current results do not necessarily reflect the role of nNOS in completely healthy humans. To minimize potential confounding effects of these factors, we studied arteries that appeared angiographically smooth and unobstructed, and we omitted medications on the day of the study. In addition, the impact of coexisting atherosclerosis risk factors in this study was not sufficient to impair endothelium-dependent vasodilation to substance P.

## GRANTS

This work was supported by British Heart Foundation Grant PG/10/53/28452 and the Department of Health via a National Institute for Health Research Biomedical Research Centre award to Guy’s & St Thomas’ National Health Service (NHS) Foundation Trust, in partnership with King’s College London and King’s College Hospital NHS Foundation Trust.

## DISCLOSURES

No conflicts of interest, financial or otherwise, are declared by the authors.

## AUTHOR CONTRIBUTIONS

S.G.K., N.M., H.S., A.R.C., K.M., F.K., and A.M.S. performed experiments; S.G.K., N.M., H.S., and K.O. analyzed data; S.G.K., N.M., P.C., and A.M.S. interpreted results of experiments; S.G.K. prepared figures; S.G.K. drafted manuscript; S.G.K., N.M., P.C., and A.M.S. edited and revised manuscript; A.M.S. conceived and designed research; all authors approved final version of manuscript.

## References

[1] Arrighi JA, Burg M, Cohen IS, Kao AH, Pfau S, Caulin-Glaser T, Zaret BL, Soufer R Myocardial blood-flow response during mental stress in patients with coronary artery disease. Lancet 356: 310–311, 2000. doi:10.1016/S0140-6736(00)02510-1. 11071190

[2] Bachetti T, Comini L, Curello S, Bastianon D, Palmieri M, Bresciani G, Callea F, Ferrari R Co-expression and modulation of neuronal and endothelial nitric oxide synthase in human endothelial cells. J Mol Cell Cardiol 37: 939–945, 2004. doi:10.1016/j.yjmcc.2004.07.006. 15522271

[3] Bassenge E, Busse R Endothelial modulation of coronary tone. Prog Cardiovasc Dis 30: 349–380, 1988. doi:10.1016/0033-0620(88)90003-5. 3279461

[4] Blumenthal JA, Jiang W, Waugh RA, Frid DJ, Morris JJ, Coleman RE, Hanson M, Babyak M, Thyrum ET, Krantz DS, O’Connor C Mental stress-induced ischemia in the laboratory and ambulatory ischemia during daily life. Association and hemodynamic features. Circulation 92: 2102–2108, 1995. doi:10.1161/01.CIR.92.8.2102. 7554188

[5] Boulanger CM, Heymes C, Benessiano J, Geske RS, Lévy BI, Vanhoutte PM Neuronal nitric oxide synthase is expressed in rat vascular smooth muscle cells: activation by angiotensin II in hypertension. Circ Res 83: 1271–1278, 1998. doi:10.1161/01.RES.83.12.1271. 9851944

[6] Burg MM, Edmondson D, Shimbo D, Shaffer J, Kronish IM, Whang W, Alcántara C, Schwartz JE, Muntner P, Davidson KW The “perfect storm” and acute coronary syndrome onset: do psychosocial factors play a role? Prog Cardiovasc Dis 55: 601–610, 2013. doi:10.1016/j.pcad.2013.03.003. 23621970 PMC3652628

[7] Capettini LS, Cortes SF, Silva JF, Alvarez-Leite JI, Lemos VS Decreased production of neuronal NOS-derived hydrogen peroxide contributes to endothelial dysfunction in atherosclerosis. Br J Pharmacol 164: 1738–1748, 2011. doi:10.1111/j.1476-5381.2011.01500.x. 21615722 PMC3230819

[8] Chauhan A, Mullins PA, Taylor G, Petch MC, Schofield PM Effect of hyperventilation and mental stress on coronary blood flow in syndrome X. Br Heart J 69: 516–524, 1993. doi:10.1136/hrt.69.6.516. 8343318 PMC1025163

[9] Dakak N, Quyyumi AA, Eisenhofer G, Goldstein DS, Cannon RO III Sympathetically mediated effects of mental stress on the cardiac microcirculation of patients with coronary artery disease. Am J Cardiol 76: 125–130, 1995. doi:10.1016/S0002-9149(99)80043-5. 7611145

[10] Deanfield JE, Halcox JP, Rabelink TJ Endothelial function and dysfunction: testing and clinical relevance. Circulation 115: 1285–1295, 2007. doi:10.1161/CIRCULATIONAHA.106.652859. 17353456

[11] Dimsdale JE Psychological stress and cardiovascular disease. J Am Coll Cardiol 51: 1237–1246, 2008. doi:10.1016/j.jacc.2007.12.024. 18371552 PMC2633295

[12] Doucette JW, Corl PD, Payne HM, Flynn AE, Goto M, Nassi M, Segal J Validation of a Doppler guide wire for intravascular measurement of coronary artery flow velocity. Circulation 85: 1899–1911, 1992. doi:10.1161/01.CIR.85.5.1899. 1572046

[13] Drexler H, Zeiher AM, Wollschläger H, Meinertz T, Just H, Bonzel T Flow-dependent coronary artery dilatation in humans. Circulation 80: 466–474, 1989. doi:10.1161/01.CIR.80.3.466. 2766503

[14] Egashira K, Katsuda Y, Mohri M, Kuga T, Tagawa T, Kubota T, Hirakawa Y, Takeshita A Role of endothelium-derived nitric oxide in coronary vasodilatation induced by pacing tachycardia in humans. Circ Res 79: 331–335, 1996. doi:10.1161/01.RES.79.2.331. 8756012

[15] Fauvel JP, Bernard N, Laville M, Daoud S, Pozet N, Zech P Reproducibility of the cardiovascular reactivity to a computerized version of the Stroop stress test in normotensive and hypertensive subjects. Clin Auton Res 6: 219–224, 1996. doi:10.1007/BF02291137. 8902318

[16] Förstermann U, Sessa WC Nitric oxide synthases: regulation and function. Eur Heart J 33: 829–837, 2012. doi:10.1093/eurheartj/ehr304. 21890489 PMC3345541

[17] Freyschuss U, Hjemdahl P, Juhlin-Dannfelt A, Linde B Cardiovascular and sympathoadrenal responses to mental stress: influence of beta-blockade. Am J Physiol Heart Circ Physiol 255: H1443–H1451, 1988. 3202206 10.1152/ajpheart.1988.255.6.H1443

[18] Han G, Ma H, Chintala R, Miyake K, Fulton DJ, Barman SA, White RE Nongenomic, endothelium-independent effects of estrogen on human coronary smooth muscle are mediated by type I (neuronal) NOS and PI3-kinase-Akt signaling. Am J Physiol Heart Circ Physiol 293: H314–H321, 2007. doi:10.1152/ajpheart.01342.2006. 17351066

[19] Ichihara A, Inscho EW, Imig JD, Navar LG Neuronal nitric oxide synthase modulates rat renal microvascular function. Am J Physiol Renal Physiol 274: F516–F524, 1998. 9530268 10.1152/ajprenal.1998.274.3.F516

[20] Khan SG, Geer A, Fok HW, Shabeeh H, Brett SE, Shah AM, Chowienczyk PJ Impaired neuronal nitric oxide synthase-mediated vasodilator responses to mental stress in essential hypertension. Hypertension 65: 903–909, 2015. doi:10.1161/HYPERTENSIONAHA.114.04538. 25733243

[21] Lefroy DC, Crake T, Uren NG, Davies GJ, Maseri A Effect of inhibition of nitric oxide synthesis on epicardial coronary artery caliber and coronary blood flow in humans. Circulation 88: 43–54, 1993. doi:10.1161/01.CIR.88.1.43. 8319355

[22] Levy AS, Chung JC, Kroetsch JT, Rush JW Nitric oxide and coronary vascular endothelium adaptations in hypertension. Vasc Health Risk Manag 5: 1075–1087, 2009. doi:10.2147/VHRM.S7464. 20057900 PMC2801631

[23] Mattson DL, Meister CJ Renal cortical and medullary blood flow responses to L-NAME and ANG II in wild-type, nNOS null mutant, and eNOS null mutant mice. Am J Physiol Regul Integr Comp Physiol 289: R991–R997, 2005. doi:10.1152/ajpregu.00207.2005. 15961532

[24] Melikian N, Kearney MT, Thomas MR, De Bruyne B, Shah AM, MacCarthy PA A simple thermodilution technique to assess coronary endothelium-dependent microvascular function in humans: validation and comparison with coronary flow reserve. Eur Heart J 28: 2188–2194, 2007. doi:10.1093/eurheartj/ehm269. 17644509

[25] Melikian N, Seddon MD, Casadei B, Chowienczyk PJ, Shah AM Neuronal nitric oxide synthase and human vascular regulation. Trends Cardiovasc Med 19: 256–262, 2009. doi:10.1016/j.tcm.2010.02.007. 20447567 PMC2984617

[26] Nabel EG, Ganz P, Gordon JB, Alexander RW, Selwyn AP Dilation of normal and constriction of atherosclerotic coronary arteries caused by the cold pressor test. Circulation 77: 43–52, 1988. doi:10.1161/01.CIR.77.1.43. 2826047

[27] Paolocci N, Pagliaro P, Isoda T, Saavedra FW, Kass DA Role of calcium-sensitive K(+) channels and nitric oxide in in vivo coronary vasodilation from enhanced perfusion pulsatility. Circulation 103: 119–124, 2001. doi:10.1161/01.CIR.103.1.119. 11136696

[28] Park SJ, Kang SJ, Ahn JM, Shim EB, Kim YT, Yun SC, Song H, Lee JY, Kim WJ, Park DW, Lee SW, Kim YH, Lee CW, Mintz GS, Park SW Visual-functional mismatch between coronary angiography and fractional flow reserve. JACC Cardiovasc Interv 5: 1029–1036, 2012. doi:10.1016/j.jcin.2012.07.007. 23078732

[29] Rochette L, Lorin J, Zeller M, Guilland JC, Lorgis L, Cottin Y, Vergely C Nitric oxide synthase inhibition and oxidative stress in cardiovascular diseases: possible therapeutic targets? Pharmacol Ther 140: 239–257, 2013. doi:10.1016/j.pharmthera.2013.07.004. 23859953

[30] Sánchez A, Contreras C, Martínez MP, Climent B, Benedito S, García-Sacristán A, Hernández M, Prieto D Role of neural NO synthase (nNOS) uncoupling in the dysfunctional nitrergic vasorelaxation of penile arteries from insulin-resistant obese Zucker rats. PLoS One 7: e36027, 2012. doi:10.1371/journal.pone.0036027. 22540017 PMC3335073

[31] Schöder H, Silverman DH, Campisi R, Karpman H, Phelps ME, Schelbert HR, Czernin J Effect of mental stress on myocardial blood flow and vasomotion in patients with coronary artery disease. J Nucl Med 41: 11–16, 2000. 10647599

[32] Seddon M, Melikian N, Dworakowski R, Shabeeh H, Jiang B, Byrne J, Casadei B, Chowienczyk P, Shah AM Effects of neuronal nitric oxide synthase on human coronary artery diameter and blood flow in vivo. Circulation 119: 2656–2662, 2009. doi:10.1161/CIRCULATIONAHA.108.822205. 19433760

[33] Seddon MD, Chowienczyk PJ, Brett SE, Casadei B, Shah AM Neuronal nitric oxide synthase regulates basal microvascular tone in humans in vivo. Circulation 117: 1991–1996, 2008. doi:10.1161/CIRCULATIONAHA.107.744540. 18391107

[34] Sequeira IM, Haberberger RV, Kummer W Atrial and ventricular rat coronary arteries are differently supplied by noradrenergic, cholinergic and nitrergic, but not sensory nerve fibres. Ann Anat 187: 345–355, 2005. doi:10.1016/j.aanat.2005.05.003. 16163847

[35] Shabeeh H, Melikian N, Dworakowski R, Casadei B, Chowienczyk P, Shah AM Differential role of endothelial versus neuronal nitric oxide synthase in the regulation of coronary blood flow during pacing-induced increases in cardiac workload. Am J Physiol Heart Circ Physiol 304: H1277–H1282, 2013. doi:10.1152/ajpheart.00927.2012. 23479261 PMC3652090

[36] Shiode N, Morishima N, Nakayama K, Yamagata T, Matsuura H, Kajiyama G Flow-mediated vasodilation of human epicardial coronary arteries: effect of inhibition of nitric oxide synthesis. J Am Coll Cardiol 27: 304–310, 1996. doi:10.1016/0735-1097(95)00465-3. 8557898

[37] Shiode N, Nakayama K, Morishima N, Yamagata T, Matsuura H, Kajiyama G Nitric oxide production by coronary conductance and resistance vessels in hypercholesterolemia patients. Am Heart J 131: 1051–1057, 1996. doi:10.1016/S0002-8703(96)90076-9. 8644581

[38] Tagawa T, Mohri M, Tagawa H, Egashira K, Shimokawa H, Kuga T, Hirooka Y, Takeshita A Role of nitric oxide in substance P-induced vasodilation differs between the coronary and forearm circulation in humans. J Cardiovasc Pharmacol 29: 546–553, 1997. doi:10.1097/00005344-199704000-00017. 9156366

[39] Toda N, Imamura T, Okamura T Alteration of nitric oxide-mediated blood flow regulation in diabetes mellitus. Pharmacol Ther 127: 189–209, 2010. doi:10.1016/j.pharmthera.2010.04.009. 20546780

[40] Topol EJ, Nissen SE Our preoccupation with coronary luminology. The dissociation between clinical and angiographic findings in ischemic heart disease. Circulation 92: 2333–2342, 1995. doi:10.1161/01.CIR.92.8.2333. 7554219

[41] Vita JA, Treasure CB, Yeung AC, Vekshtein VI, Fantasia GM, Fish RD, Ganz P, Selwyn AP Patients with evidence of coronary endothelial dysfunction as assessed by acetylcholine infusion demonstrate marked increase in sensitivity to constrictor effects of catecholamines. Circulation 85: 1390–1397, 1992. doi:10.1161/01.CIR.85.4.1390. 1555281

[42] Wei J, Rooks C, Ramadan R, Shah AJ, Bremner JD, Quyyumi AA, Kutner M, Vaccarino V Meta-analysis of mental stress-induced myocardial ischemia and subsequent cardiac events in patients with coronary artery disease. Am J Cardiol 114: 187–192, 2014. doi:10.1016/j.amjcard.2014.04.022. 24856319 PMC4126399

[43] Yeung AC, Vekshtein VI, Krantz DS, Vita JA, Ryan TJ Jr, Ganz P, Selwyn AP The effect of atherosclerosis on the vasomotor response of coronary arteries to mental stress. N Engl J Med 325: 1551–1556, 1991. doi:10.1056/NEJM199111283252205. 1944439

[44] Yoshida K, Okamura T, Kimura H, Bredt DS, Snyder SH, Toda N Nitric oxide synthase-immunoreactive nerve fibers in dog cerebral and peripheral arteries. Brain Res 629: 67–72, 1993. doi:10.1016/0006-8993(93)90482-3. 7506984

[45] Zeiher AM, Drexler H, Wollschlaeger H, Saurbier B, Just H Coronary vasomotion in response to sympathetic stimulation in humans: importance of the functional integrity of the endothelium. J Am Coll Cardiol 14: 1181–1190, 1989. doi:10.1016/0735-1097(89)90414-2. 2808971

